# P-smoother: efficient PBWT smoothing of large haplotype panels

**DOI:** 10.1093/bioadv/vbac045

**Published:** 2022-06-20

**Authors:** William Yue, Ardalan Naseri, Victor Wang, Pramesh Shakya, Shaojie Zhang, Degui Zhi

**Affiliations:** School of Biomedical Informatics, University of Texas Health Science Center at Houston, Houston, TX 77030, USA; School of Biomedical Informatics, University of Texas Health Science Center at Houston, Houston, TX 77030, USA; School of Biomedical Informatics, University of Texas Health Science Center at Houston, Houston, TX 77030, USA; Department of Computer Science, University of Central Florida, Orlando, FL 32816, USA; Department of Computer Science, University of Central Florida, Orlando, FL 32816, USA; School of Biomedical Informatics, University of Texas Health Science Center at Houston, Houston, TX 77030, USA

## Abstract

**Motivation:**

As large haplotype panels become increasingly available, efficient string matching algorithms such as positional Burrows-Wheeler transformation (PBWT) are promising for identifying shared haplotypes. However, recent mutations and genotyping errors create occasional mismatches, presenting challenges for exact haplotype matching. Previous solutions are based on probabilistic models or seed-and-extension algorithms that passively tolerate mismatches.

**Results:**

Here, we propose a PBWT-based smoothing algorithm, P-smoother, to actively ‘correct’ these mismatches and thus ‘smooth’ the panel. P-smoother runs a bidirectional PBWT-based panel scanning that flips mismatching alleles based on the overall haplotype matching context, which we call the IBD (identical-by-descent) prior. In a simulated panel with 4000 haplotypes and a 0.2% error rate, we show it can reliably correct 85% of errors. As a result, PBWT algorithms running over the smoothed panel can identify more pairwise IBD segments than that over the unsmoothed panel. Most strikingly, a PBWT-cluster algorithm running over the smoothed panel, which we call PS-cluster, achieves state-of-the-art performance for identifying multiway IBD segments, a challenging problem in the computational community for years. We also showed that PS-cluster is adequately efficient for UK Biobank data. Therefore, P-smoother opens up new possibilities for efficient error-tolerating algorithms for biobank-scale haplotype panels.

**Availability and implementation:**

Source code is available at github.com/ZhiGroup/P-smoother.

## 1 Introduction

In the biobank era, large collections of whole-genome genotype data are becoming abundantly available. Modern phasing methods (Delaneau *et al.*, 2019; Loh *et al.*, 2016a, b) can generate accurate large haplotype panels. On the surface, such panels are large matrices of binary values. However, these values are generated by evolutionary processes at the crude scale and by genealogical processes at the fine scale. It is important to model haplotype panels with these processes in mind.

The traditional phylogenetic or population genetics modeling methods typically view haplotype panels as a collection of independent columns, each representing a polymorphic variant site. The Li-and-Stephens model (Li and Stephens, 2003) is a hidden Markov model capturing the dependencies of adjacent sites. However, it is inefficient for modeling long haplotypes. The positional Burrows-Wheeler transformation (PBWT; Durbin, 2014) views a haplotype panel as a collection of aligned sequences. By sorting these sequences at each site according to their reverse prefix, PBWT enables efficient exact matching of aligned substrings in haplotype panels. As a result, PBWT has served as an engine for efficient solutions for identical-by-descent (IBD) segment matching (Freyman *et al.*, 2021; Naseri *et al.*, 2019; Zhou *et al.*, 2020), runs-of-homozygosity cluster calling (Naseri *et al.*, 2020) and genotype imputation (Browning *et al.*, 2018; Rubinacci *et al.*, 2020) for very large haplotype panels.

One major problem for PBWT in modeling real-world sequences is that real data typically contain mismatches disrupting long haplotype matches. The major source of genotyping errors is from genotype calling pipelines. Despite advanced genotype calling methods, genotyping errors still exist and the rate can vary between 0.1% and 0.5%. For example, 0.1% genotyping error is expected in sequencing data (Wang *et al.*, 2021) while array data from Wellcome Trust Case Control Consortium (WTCCC) has an estimated error rate of 0.2% (Marchini and Howie, 2010). In addition, phasing methods may introduce additional mismatches in haplotypes. Even without errors, rare and private mutations and somatic mosaicisms (Loh *et al.*, 2018) exist naturally. Each of these factors has the potential of creating mismatches that make PBWT ‘bristle’, i.e. with fragmented long matches. Furthermore, it can be difficult to discern these sources of mismatches from each other.

Existing PBWT-based IBD segment calling methods have formerly introduced methods for tolerating mismatches. RaPID (Naseri *et al.*, 2019) introduced a probabilistic model over multiple random projections of a panel to tolerate mismatches. hap-IBD (Zhou *et al.*, 2020) used a ‘seed-and-extension’ method that extends the matches seeded by short exact PBWT matches. These methods treat mismatches as random errors, thus can only passively ‘tolerate’ them.

In this work, we propose an IBD prior model capturing the continuity of matching haplotypes. For a haplotype sequence value at a particular site, our model encodes the information of sequences IBD to this sequence at the surrounding sites. While not in an explicit probabilistic form, the IBD prior can be used as a reference against which the actual observed haplotype value can be checked. Based on the IBD prior, we introduce a PBWT-based smoothing process called PBWT-smoother, or P-smoother, that can actively correct mismatches in haplotype panels. P-smoother leverages the bidirectional PBWT (bi-PBWT; Naseri *et al.*, 2021) data structure and scanning algorithm to efficiently make corrections.

We evaluated the effectiveness of P-smoother for correcting errors and mutations to establish its effectiveness. We then applied standard PBWT algorithms to the smoothed haplotype panels and identified multiway IBD segments, or IBD clusters. Interestingly, to the best of our knowledge, there are no efficient methods for identifying IBD clusters in large haplotype panels.

Overall, our contributions are the following. First, we developed the first IBD prior model for haplotype panels. Second, we developed a mismatch correction method that can alleviate the problem of mismatches in haplotype panels. Third, we developed a new IBD cluster calling method that is the first efficient method for large haplotype panels.

## 2 Methods

### 2.1 The IBD prior model

The underlying idea of the IBD prior model is that at any *x_ik_* position, indicating the *i*-th sequence at column *k* of a haplotype panel $x\in {\left\{0,1\right\}}^{M\times N}$, the nucleotide sequence value *x_ik_* is typically consistent with other sequences *x_j_* that are IBD with this sequence *x_i_*. Specifically, we define *x_ik’_*s *L*-block to its left, $B_{-}(x_{ik},L)$, as the maximal set of haplotypes such that for all $x_{j}\in B_{-}(x_{ik},L)$, *x_ja_* = *x_ia_* for k−L≤a<k. Similarly, its *L*-block to its right, $B_{+}(x_{ik},L)$, is defined as the maximal set of haplotypes such that for all $x_{j}\in B_{+}(x_{ik},L)$, *x_ja_* = *x_ia_* for k<a≤k+L. When not ambiguous, we refer to them as $B_{-}$ and $B_{+}$. $B_{-}$ and $B_{+}$ represent the set of sequences that are identical-by-state (IBS) to the left and to the right of site *k* for sequence *x_i_*. Furthermore, both $B_{-}$ and $B_{+}$ likely contain sequences IBD to *x_i_*. Therefore, by way of transitivity, most sequences should be shared between $B_{-}$ and $B_{+}$ because recombination events are expected to be rare at site *k*. Here, we define the set of haplotypes that are IBS to sequence *x_i_* at position *k*, without consideration of position *k* itself, $B(x_{ik},L)=B_{-}(x_{ik},L)\cap B_{+}(x_{ik},L)$, as the *bidirectional IBD set* or the *bidirectional block* of *x_ik_*. The rationale of having a bidirectional match is that it will filter out non-IBD one-sided *L*-blocks that are unlikely to continue on the other side, allow us to put column *k* into the center of focus and surround matching sequences with enough sites to help cushion against potential edge effects.

Focusing on position *k* itself, the location *x_ik_* has a *reference set* $R_{ik}(L)=\{x_{jk}|j\in B(x_{ik},L)\}\backslash \{x_{ik}\}$. Without directly observing the value of *x_ik_*, we can infer the value of *x_ik_* using its reference set. We can therefore define an IBD prior function *π* summarizing the information in $R_{ik}(L)$. For example, we can introduce three criteria:

1. The local haplotype match ($B_{-}$ and $B_{+}$) length *L* is large.
2. The number of sequences in $R_{ik}(L)$ is large: $|R_{ik}(L)|\geq W$.
3. A vast majority of the sequences in $R_{ik}(L)$ agree: the allele frequency *AF*(*b*), where *b* is the minor allele, is below a certain threshold *ρ*.

Formally, we can define the prior as a simple rule-based function with parameter θ=(L,W,ρ):

(1) $$\begin{array}{c} \pi_{\theta }(b,x,i,k)=Pr(x_{ik}=b|L,W,\rho )\\ =\{\begin{array}{cc} 0, & |R_{ik}(L)|\geq W\;\text{and}\;AF(b)<\rho \\ 1, & \text{otherwise}. \end{array} \end{array}.$$

If the probability is 1 for both *b *=* *0 and *b *=* *1, i.e. the reference set $R_{ik}(L)$ is not large enough, then we keep the original allele value due to a lack of conclusive evidence. Certainly, more sophisticated priors could be designed that better capture the population genetics process and the sampling process. In this work, we will be primarily experimenting with this simple prior and leave more systematic investigation of other priors as future work.

This model can be used for genotype imputation, genotyping error correction and smoothing. For genotype imputation, the premise is that not all sequences are observable at the position *k*, and the goal is to fill in the unobserved allele values. For genotyping error correction, even though all sequences are observed, we compare the observed *x_ik_* to the estimated from Equation (1). However, as it is often difficult to distinguish errors from rare and private mutations, pure correction of errors may not be a practical goal. However, for the sake of identifying IBD blocks that might otherwise be interrupted by mismatches, it is possible to conduct a smoothing procedure. The goal of smoothing is to maximize the agreement at site *k* between *x_i_* and $B(x_{ik},L)$, i.e. maximize the overlap between $B_{-}(x_{ik},L)$ and $B_{+}(x_{ik},L)$. Thus, a natural choice is to force the correction of $x_{ik}={\text{argmax}}_{b}[\pi_{\theta }(b,x,i,k)]$. Note that for smoothing to be conducted, the number of sequences does not need to be large, as we are more interested in the continuity of the blocks $B_{-}(x_{ik},L)$ and $B_{+}(x_{ik},L)$ than in making the corrected genotype calls. Also, the smoothing parameters θ=(L,W,ρ) can be adjusted according to the objective of the downstream task. In this work, we will be mostly focusing on calling multiway IBD segments, which we attempted to optimize.

As will be described below, the design of the IBD prior is based on the availability of efficient algorithms for computing it at each position within the panel.

### 2.2 Efficient computation of the IBD smoothing through bi-PBWT

#### 2.2.1 Positional Burrows-Wheeler transformation

The underlying idea of the PBWT is to sort haplotype sequences at each site by their reversed prefixes. This allows the efficient computation of match length (longest common prefix) between adjacent haplotypes in sorted order, which we then use to efficiently detect blocks of matching haplotypes. Furthermore, PBWT can be extended to be bidirectional in order to allow for the detection of blocks across a gap (a contiguous segment of sites).

Given a haplotype panel **x**, PBWT calculates two additional arrays, the positional prefix and divergence arrays, which facilitate an efficient approach to finding long matches. The positional prefix array stores the haplotype indices in reversed prefix order at variant site *k*, while the divergence array at the variant site *k* stores the starting position of the match between each haplotype and its preceding haplotype in the reversed prefix order.

By sorting the haplotype sequences in reversed prefix order at each site *k*, each haplotype will be placed adjacent to the haplotype with which it has the longest match. A block of matching haplotypes is defined as a width-maximal set of haplotypes matching over sites [k−L,k) (Naseri *et al.*, 2020). Blocks of matching haplotypes will be bounded by a haplotype whose divergence value is greater than *k—L*, where *L* denotes the desired length of a match. Since matching haplotypes appear contiguously in the positional prefix order, the blocks of matching haplotypes can be efficiently computed. PBWT has already been used in other works to find matching blocks in large haplotype panels (Alanko *et al.*, 2019; Williams and Mumey, 2020).

#### 2.2.2 Bidirectional PBWT

Bi-PBWT (Naseri *et al.*, 2021) calculates the PBWT arrays for both the forward and reverse directions at each variant site *k*. Given a minimum cut-off length *L*, the matching blocks can be efficiently computed for both directions. We define the element-wise intersection of a forward block set *S* and a reverse block set *T* to be U(S,T)={s∩t|s∈S∧t∈T}∖{∅}, which can be computed in linear time. Most importantly, *S* and *T* do not have to be from the same site *k*. This property allows for the introduction of a gap where *S* is taken from site *k* and *T* is taken from site *k *+* g*, where *g* is the size of the gap. For memory efficiency, bi-PBWT can be implemented by first saving the reverse PBWT onto the disk prior to performing a forward scan to element-wise intersect the forward and reverse PBWTs (Naseri *et al.*, 2021).

The bi-PBWT forwarding scanning algorithm to element-wise intersect *S* and *T* at each site *k* is implemented with a radix sort. Each haplotype is assigned a forward block ID and a reverse block ID. When the haplotypes are sorted based on these IDs, haplotypes in the same forward and reverse block appear in the sorted haplotype list as a contiguous segment, which allows for the easy extraction of bidirectional blocks B∈U(S,T).

#### 2.2.3 P-smoother

**Fig. 1.**
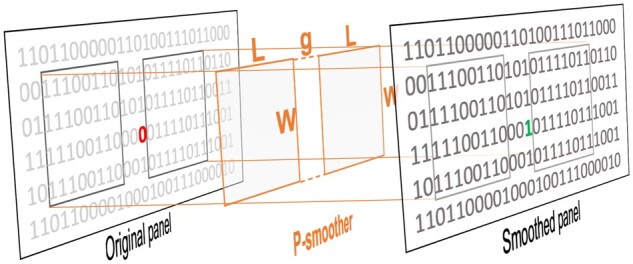
P-smoother correcting an allele in the original panel to create a smoothed panel. P-smoother utilizes the two matching blocks ($B_{-}$ and $B_{+}$) from bi-PBWT. The gap size *g* and other P-smoother parameters are highlighted. A further description of these parameters can be found in Section. The values of the parameters in this figure are L=8 sites, W=4 haplotypes, g=3 sites, ρ=35%. The MAF of the three gap sites from left to right are 50%, 0% and 25%. In order for a site in the gap to be corrected, the site must have a nonzero minimum allele frequency that is less than *ρ*. Since ρ=35% in this figure, only the third site in the gap meets the criteria to be corrected

We hypothesize that mismatches fragmenting otherwise perfect matching blocks *B* are likely to still be surrounded by fragments of smaller matching blocks $B_{-}$ and $B_{+}$. The need to identify mismatches by neighboring matching blocks on both sides of a site naturally leads us to use the bi-PBWT algorithm. bi-PBWT allows us to efficiently find bidirectional blocks that match across a gap. For the bidirectional blocks that we find using bi-PBWT, we can mismatch-correct sites inside the gap as we know mismatches inside this gap are fragmenting what would be a larger matching block. To mismatch-correct a site within the gap in a bi-PBWT block, we compute the minor allele frequency (MAF) of the alleles in the block at that site and then change the minor alleles to the major allele if the MAF is below the threshold *ρ*. Smoothing a panel with P-smoother guarantees that mismatches (below threshold *ρ*) with adjacent matching blocks $B_{-}$ and $B_{+}$ will be corrected. Note that mismatches located on the *L* sites at either end of the chromosome will not be corrected since these mismatches will lack either $B_{-}$ or $B_{+}$. However, this edge case affects a negligible number of sites as the optimal value of *L* is around 20 sites (see Section 3.4 for more details on the tuning of *L*). Figure 1 illustrates the idea of utilizing P-smoother to generate a new smoothed panel from an original panel potentially plagued by mismatches. Algorithm 1 outlines the pseudocode for mismatch-correcting inside the gap of a block B∈U found by bi-PBWT that meets the length (*L*) and width (*W*) requirements (criteria 1 and 2).

**Algorithm 1** Mismatch correct inside the gap of a block B∈U

1: **procedure**Smooth(Block *B*)

2:  zero←[],one←[] ▹ zero[i] and one[i] store the number of zeros and ones at site *i* in the gap

3:  **for**k←1**to** *G* **do**

4:   **if**min(zero[k],one[k])≤|B|·ρ**then**

5:    **if**zero[k]>one[k]**then**

6:     correctAllele←0

7:    **else**

8:     correctAllele←1

9:    **end if**

10    **for all**$x_{i}\in B$**do**

11:     $x_{ik}=\mathrm{correctAllele}$

12:    **end for**

13:   **end if**

14:  **end for**

15: **end procedure**

#### 2.2.4 P-smoother parameters

P-smoother takes four parameters: L, W, g, ρ (the first three can be found in Fig. 1). The parameters *L* (in units of sites) and *W* (in units of haplotypes) specify the minimum length and width requirements for bidirectional blocks. *g* (in units of sites) specifies the gap size of bidirectional blocks. Finally, *ρ* (a percentage) specifies the minimum allele frequency threshold for mismatches to be corrected inside of a gap. A thorough analysis and tuning of these parameters can be found in Section 3.4.

### 2.3 Time and space complexity

As P-smoother implements bi-PBWT, its time complexity is also *O*(*MN*) where *M* is the number of haplotypes and *N* is the number of sites in the panel. At any point in time, the only memory required by P-smoother is the storage of the alleles in the gap, and because there can be at most *gM* alleles in the gap, the space complexity is *O*(*gM*). A linear time complexity and sublinear space complexity with respect to the input size (*MN*) allows P-smoother to be resource efficient and highly scalable to biobank data.

### 2.4 Multiway IBD segment calling

Multiway IBD segments (i.e. IBD segments shared by multiple individuals), or IBD clusters for short, are of interest to family studies and disease mapping. Assuming equal length, IBD clusters present a stronger statistical signal than pairwise IBDs for tracing distant relatives and pedigree (Gusev *et al.*, 2011; Moltke *et al.*, 2011; Qian *et al.*, 2014). Moreover, IBD clusters can reveal long haplotypes that are signals for selection, or directly serve as inputs for association studies with phenotypes.

However, there are not many existing methods for inferring IBD clusters. The few existing methods are limited in terms of speed and the types of IBD clusters they can detect. Moltke *et al.* (2011) directly infer IBD clusters using a Markov Chain Monte Carlo method, but it is not efficient for large samples. DASH (Gusev *et al.*, 2011), EMI (Qian *et al.*, 2014) and IBD-groupon (He, 2013) postprocess pairwise IBD results to identify dense clusters of pairwise IBD segments. However, their performance heavily relies on the quality and speed of the initial pairwise IBD segment detection. Recently, consensus PBWT (cPBWT; Naseri *et al.*, 2020) has enabled directly calling PBWT blocks as IBD clusters, but it is unable to tolerate mismatches.

We propose to conduct IBD cluster calling by utilizing P-smoother to produce a smoothed haplotype panel for cPBWT to extract clusters. Our proposed new P-smoother-cluster method, or PS-cluster method for short, can directly identify IBD clusters. Of note, there are two user-specified parameters for the cPBWT algorithm, namely the minimal cluster length $L_{\min}$, the minimal cluster width $W_{\min}$, in addition to the parameters for the P-smoother algorithm. $L_{\min}$ and *L* are not necessarily the same, and neither are $W_{\min}$ and *W*.

While P-smoother and cPBWT are conceptually separate, they can be implemented simultaneously in the same pass. The reverse PBWT blocks are first computed in a reverse pass, then the forward pass is implemented with each site first being processed by P-smoother and then by cPBWT.

### 2.5 Benchmarking using simulated data

#### 2.5.1 Simulation

We simulated 1 million haplotypes of European ancestry using stdpopsim. The HapMap genetic map (GRCh37) was used to simulate haplotypes of chromosome 20. We used the population model OutOfAfrica_2T12 defined in stdpopsim and generated 500 000 Europeans. The sites with low allele frequencies (MAF < 0.1) were filtered out using bcftools. The first 4000 haplotypes were extracted for benchmarking pairwise IBD segment detection and IBD cluster detection.

#### 2.5.2 Performance evaluation for IBD cluster detection

As there is no existing method efficient enough to identify IBD clusters in large panels, we only benchmarked PS-cluster against plain cPBWT.

Interestingly, there has been little work in defining ground truth and benchmarking methods for IBD cluster detection. For example, IBDgroupon was only benchmarked for clusters of 3–5 haplotypes among a sample of 100 haplotypes and was using random sequences rather than sequences resulting from population genetic processes. Therefore, we developed our own framework for evaluating IBD cluster detection methods for large panels.

##### 2.5.2.1 Extraction of ground truth IBD clusters

We used the msprime package to generate a tree sequence, from which we extracted the IBD segments using the tskit package (Kelleher *et al.*, 2016). We created an interim panel consistent with the ground truth IBD segments by implanting identical segments for haplotypes sharing a ground truth IBD segment. Specifically, we first initialized a panel $x={\left\{-1\right\}}^{M\times N}$. As we iterated over every site *k* in a given IBD segment between haplotypes *x_i_* and *x_j_*, we updated the entries *x_ik_* and *x_jk_* in the interim panel. If both entries were –1, we randomly chose 0 or 1 to assign to both *x_ik_* and *x_jk_*. If either entry was 0 or 1, we assigned its value to the other entry. Finally, we ran cPBWT on the interim panel to extract the ground truth IBD clusters.

##### 2.5.2.2 Power and accuracy metrics

We define the area of an IBD cluster to be the number of haplotypes in the cluster multiplied by the number of sites in the cluster. Similarly, the overlap between two IBD clusters is defined as the number of shared haplotypes multiplied by the number of shared sites.

The power of a ground truth IBD cluster is defined as the maximum overlap between the ground truth IBD cluster and a reported IBD cluster, normalized by the area of the ground truth IBD cluster. The overall power is defined as the average power of the ground truth IBD clusters.

The accuracy of a reported IBD cluster is defined as the maximum overlap between the reported IBD cluster and a ground truth IBD cluster, normalized by the area of the reported IBD cluster. The overall accuracy is defined as the average accuracy of the reported IBD clusters.

## 3 Results

Here, we first showcase P-smoother’s ability to error correct before demonstrating P-smoother’s applicability to IBD segment and IBD cluster detection. Error correction and IBD/cluster detection experiments were all benchmarked on a simulated panel with 4000 haplotypes and 47 821 variant sites. P-smoother’s parameters were set to the default of L=20 sites, g=1 site, ρ=0.05 for all experiments. In the case of error correction and IBD detection, the default W=20 haplotypes was used. In the case of IBD cluster detection, *W* was set to the target cluster width $W_{\min}$ ($W=W_{\min}$).

### 3.1 Error correction

As shown in Table 1, P-smoother with default parameters of L=20 sites, W=20 haplotypes, g=1 site, ρ=0.05  can correct 84–87% of errors. P-smoother also corrected about 41 000–42 000 alleles that were not genotyping errors, which corresponds to about 0.02% of all alleles. Although rare mutations and genotyping errors are often indistinguishable, we observed that 36 459 of the nonerror corrections are shared by all four error rates (0–0.2%), leading us to believe the vast majority of these nonerror corrections are of mutations. As such, the precision values in Table 1 serve as a lower bound and are likely a significant underestimate, since the primary goal of P-smoother is to smooth mismatches and not purely to correct errors. In an attempt to more accurately represent P-smoother’s smoothing ability, we treat the 36 459 shared nonerrors corrected as rare mutations and combine them with true errors into a single category known as mismatches. Under this benchmark, P-smoother achieves 87–93% recall and 93–98% precision over error rates of 0.05–0.2%, indicating that P-smoother would significantly reduce mismatch rates and thus facilitate downstream haplotype matching tasks.

**Table 1.** Error correction results

Error rate	Errors	Corrections	Errors corrected	Nonerrors corrected	Recall	Precision
0%	0	42,150	0	42,150	NA	0%
0.05%	47,572	83,304	41,377	41,927	86.98%	49.67%
0.10%	96,260	124,118	82,621	41,497	85.83%	66.57%
0.20%	190,840	201,543	160,505	41,038	84.10%	79.64%

### 3.2 P-smoother improves power of detecting pairwise IBD segments

**Fig. 2.**
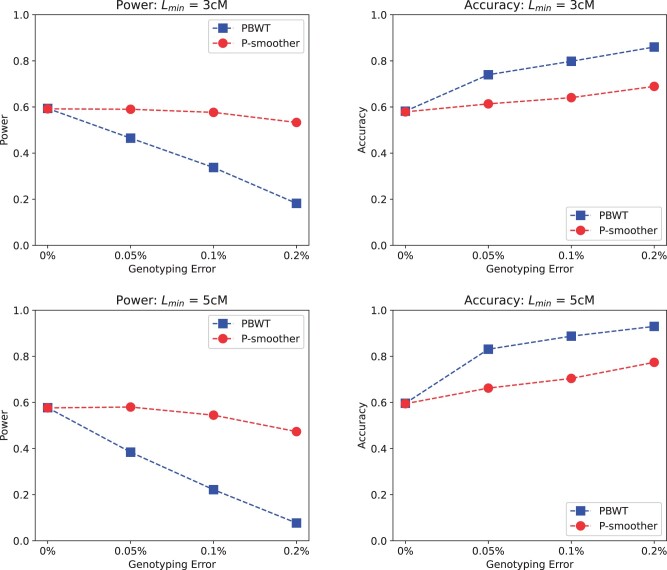
Power and accuracy of pairwise IBD segment detection with and without smoothing. The detection power of PBWT without smoothing is significantly lower in panels with genotyping errors

Figure 2 compares the IBD segment detection of PBWT versus P-smoother for the target lengths 3 and 5 cM. The power and accuracy trends for both target IBD segment lengths are similar. When subjected to increasing genotyping error, P-smoother is able to hold its detection power relatively stable while the detection power of PBWT drops significantly. With regards to detection accuracy, PBWT and P-smoother both show a steady increase in accuracy when subjected to increasing genotyping error. While PBWT does increase in accuracy at a slightly faster rate than P-smoother, PBWT’s higher accuracy is not significant when its power is approaching the single-digit percentages in most cases.

Please note that while P-smoother offers an improvement to PBWT for IBD segment detection, we do not claim to be the state-of-the-art in IBD segment detection. P-smoother’s forte is still multiway IBD cluster detection, but we seek to demonstrate that a panel smoothed by P-smoother offers improvements to not one but multiple potential downstream analyses.

### 3.3 P-smoother followed by PBWT-cluster deliver state-of-the-art multiway IBD cluster detection

**Fig. 3.**
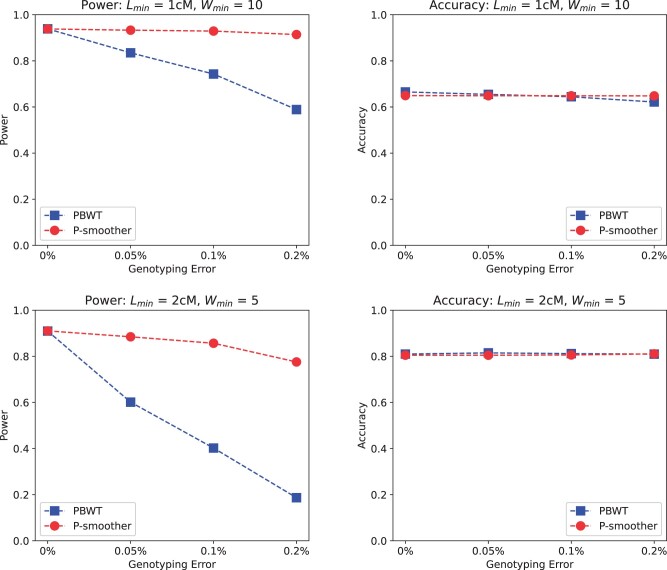
Power and accuracy of IBD clusters detection with and without smoothing. PBWT without smoothing has a low detection power in panels with genotyping errors. P-smoother has significantly higher power and near-identical accuracy for minimum lengths of 1 and 2 cM

Figure 3 compares the multiway IBD cluster detection of PBWT versus P-smoother for two targets. The first multiway IBD cluster target is $L_{\min}=1\;\text{cM},\;W_{\min}=10\;\text{haplotypes}$ and the second is $L_{\min}=2\;\text{cM},\;W_{\min}=5\;\text{haplotypes}$. When subjected to increasing genotyping error, the accuracy of both methods is able to remain constant. With regards to power, however, P-smoother is able to hold its power relatively stable when subjected to increasing genotyping error while PBWT’s power drops significantly.

The conclusively superior multiway IBD cluster detection ability of P-smoother compared to PBWT (the current state-of-the-art) establishes that P-smoother elevates state-of-the-art multiway IBD detection to an optimal level that will bring impactful improvements to downstream multiway IBD cluster analyses.

### 3.4 Parameter tuning

With the goal of optimizing P-smoother for IBD cluster detection, most of the tuning was performed on the IBD cluster detection task. Through parameter tuning, we found that the optimal default parameters for IBD cluster detection are $L=20\;\text{sites},\;W=W_{\min},\;g=1\;\text{site},\;\rho =0.05$ (see GitHub for tuning details). For error correction, the default parameters remain the same except W=20 haplotypes. For all tuning experiments, parameters that were not being tuned were left at the default values.

As we increased the parameter *L*, we observed a trend of increasing accuracy and decreasing power. This is expected since longer $B_{-}$ and $B_{+}$ blocks surrounding an allele indicate a higher probability of a mismatch. As we varied *L* from 10 to 30 sites, the accuracy only increased by 0.2% while the power only decreased by 0.4%.

As we increased the parameter *ρ*, we observed a trend of decreasing accuracy and increasing power. This is anticipated since a greater *ρ* allows corrections to happen at a lower confidence level and allows more mismatches to be corrected per block. As we varied *ρ* from 1% to 10%, we saw a 0.9% decrease in accuracy and a 3% increase in power.

As we increased the parameter *g*, we saw a trend of decreasing power and accuracy. This is expected since blocks $B_{-}$ and $B_{+}$ that are further apart give a weaker signal as to the true values of alleles in the gap between. As we varied *g* from 1 to 5 sites, we saw a 1.1% decrease in accuracy and a 0.2% decrease in power.

For IBD cluster detection, we found it clear to set $W=W_{\min}$. For error detection, as we increased the parameter *W*, we saw a trend of decreasing recall and increasing precision. This is expected since a larger minimum block width provides a larger sample size of alleles, increasing the confidence in the IBD prior’s predicted alleles.

A potential concern is that P-smoother’s parameters are hypersensitive to small changes. However, as shown in the above analysis, the parameters are all robust to slight adjustments.

### 3.5 UK biobank results

The benchmarks below showcase P-smoother’s and PS-cluster’s performance on real biobank-scale data, namely UK Biobank chromosome 20 consisting of 974 818 haplotypes and 17 197 variant sites. We ran the benchmarks on an 8-core 2.10 GHz Intel Xeon E5-2620 v4.

To examine P-smoother’s potential to improve pairwise IBD segment detection, we ran P-smoother on UK Biobank chromosome 20 to compare the original and smoothed panels. P-smoother took 3.53 CPU hours and 256 MB memory. In each panel, we removed the first 50 haplotypes to query against the rest of the haplotypes for IBD segments spanning at least 5 cM. In the original panel, 10 033 matches were reported, whereas in the smoothed panel, 11 647 matches were reported, for a 16% increase. To legitimize the greater number of matches in the absence of a definitive IBD segment ground truth, we ran RaPID, a top IBD segment detection tool for biobank-scale data that leverages random projections along with PBWT (Naseri *et al.*, 2019), on the original panel with a match length requirement of 5 cM. Haplotypes between which RaPID reported a match were considered ground truth IBD. For each panel, we computed the percentage of reported IBD segments that corresponded to a ground truth IBD segment. The original panel yielded 99.3%, and the smoothed panel yielded 97.6%, indicating that P-smoother retains high accuracy while offering more powerful IBD segment detection.

To examine P-smoother’s potential to improve IBD cluster detection, we ran P-smoother on chromosome 20 from UK Biobank. PS-cluster took 3.74 CPU hours and 276 MB memory. PS-cluster identified 3 656 950 100-way clusters over 1 cM. Due to the lack of efficient benchmarking for IBD clusters, this metric simply gives a rough portrayal of PS-cluster’s power.

## 4 Discussions

In this work, we presented the concept of an IBD prior as well as efficient algorithms for computing our IBD prior in large haplotype panels. We showed that our IBD prior is able to produce a smoothed hapotype panel in which mismatches between otherwise matching haplotypes are corrected. A direct effect of this smoothing procedure is that the length of haplotype matches is increased. We demonstrated through experiments with simulated and real data that P-smoother boosted the detection power of pairwise IBD segments and IBD clusters with little loss of accuracy.

The theoretical contribution of this work is our new IBD prior, which helps identify the discontinuities in haplotype matches. Unlike the traditional probabilistic Li-and-Stephens model, which captures a large degree of uncertainty in combining short haplotypes, our model is inspired by the efficient PBWT data structure for long haplotype matching. Noticeably, our model adopts the ‘algorithm first’ philosophy rather than the ‘probability theory first’ philosophy, hence the simplicity of our IBD prior. Our model’s high scalability makes it future-proof as biobanks with millions of samples become commonly available.

P-smoother is the first approach of error correction in haplotype panels. While error correction has been common practice in cleaning up noisy measurements in, for example, NGS data (Mitchell *et al.*, 2020), P-smoother is applied to the already highly optimized haplotype panels. Therefore, a potential concern is that we might ‘over-correct’ the errors by also flipping some real mutations. However, Sections and show that P-smoother is useful for tasks not sensitive to rare mutations, such as IBD segment and IBD cluster detection. To make P-smoother useful for downstream tasks that rely on rare variants, one could postprocess the matches in the smoothed panel by cross-referencing with the original panel. However, as distinguishing between errors and mutations is often difficult, we leave it as future research. Given that we are not attempting to distinguish errors from recent mutations, the ‘Non-errors Corrected’ column in Table 1 misrepresents the false positives. In order to give a lower bound, the precision values in Table 1 were computed under the assumption that there are no mutations and that all of the nonerror corrections were miscorrections. This hypothetical assumption clearly leads the precision values to be considerable underestimates.

Reassuringly, P-smoother has been shown to have robust parameters (*L*, *W*, *g*, *ρ*) and to be robust against error rates from 0% to 0.2%. Moreover, P-smoother’s scalability is reinforced by benchmarks on panels ranging from 4000 to 1 million haplotypes.

Perhaps, the best practical results of P-smoother are in IBD cluster detection task. Before now, there did not exist a method for identifying IBD clusters that could scale to thousands of haplotypes. PS-cluster, powered by P-smoother, offers state-of-the-art IBD cluster results: ∼80% power and 80% accuracy for 2 cM-long clusters of over 5 haplotypes, or 90% power and 65% accuracy for 1 cM-long clusters of over 10 haplotypes. These results should suffice to enable IBD mapping in large cohorts. A rigorous comparison of available clustering methods regarding different evaluation metrics is beyond the scope of this paper, and we aim to address this issue in future works.

As additional future works, P-smoother’s parameters can be optimized for scenarios with various error rates, sample sizes and demographic histories. Our IBD prior model could be applied to genotype imputation or be made more sophisticated to give more accurate predictions and differentiate between errors and mutations. Furthermore, cross-referencing the smoothed panel with the original panel could allow for further refinement of reported matches.

## Funding

This work was supported by the National Institutes of Health grants R01HG010086, R56HG011509 and OT2OD002751. This research has been conducted using the UK Biobank Resource under Application Number 24247.

*Conflict of Interest*: none declared.

## Data Availability

The data underlying this article are available in the article.
